# Age and Daily Positive Events Moderate the Link Between Depressive Symptoms and Inflammation

**DOI:** 10.1093/geroni/igaa057.1620

**Published:** 2020-12-16

**Authors:** Jin Wen, David Almeida, Nancy Sin

**Affiliations:** 1 University of British Columbia, Vancouver, British Columbia, Canada; 2 Pennsylvania State University, University Park, Pennsylvania, United States

## Abstract

Inflammation is a pathway underlying numerous aging-related conditions. Depression is related to elevated inflammation, whereas daily positive events have been linked to lower inflammation; these psychological experiences may interact with age to predict inflammation. The purpose of this study was to examine whether daily positive events moderate the association between depressive symptoms and inflammation in an adult lifespan sample. A sample of 343 adults ages 25-75 (55% women, 83% white) in the Midlife in the United States Refresher Study completed daily diary interviews for 8 evenings about their daily positive events. Depressive symptoms were assessed with the 20-item Center for Epidemiological Studies Depression scale, and blood samples were assayed for inflammatory markers interleukin-6 (IL-6) and C-reactive protein (CRP). On average, depression scores ranged from 0 to 44 (mean = 9.31, SD = 7.80), and participants reported 1.25 (SD = .70) positive events per day (range = 0–5). Depressive symptoms and daily positive events were separately associated with higher and lower log IL-6 and CRP, respectively. Depressive symptoms, daily positive events, and age interacted such that daily positive events predicted lower IL-6 (but not CRP) among midlife and older adults who reported lower depressive symptoms, whereas positive events were not related to inflammation among younger adults. Thus, these findings suggest that the protective association between daily positive events and inflammation was blunted when depressive symptoms were elevated and for younger adults. This work has implications for understanding age variations in the role of positive experiences in depression and inflammation.

